# Sutural cataract

**DOI:** 10.11604/pamj.2020.36.34.18792

**Published:** 2020-05-26

**Authors:** Hassan Moutei, Meriem Abdellaoui

**Affiliations:** 1 Department of Ophthalmology, University Hospital Hassan ll, Fez, Morocco

**Keywords:** Cataract, congenital, amblyopia

Domain: Ophthalmology

## Image in medicine

**Figure 1:**
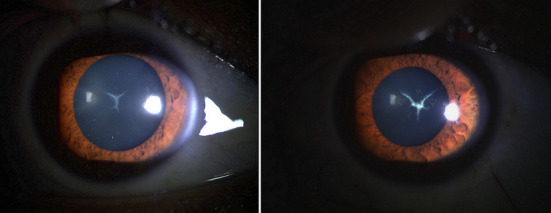
Slit lamp photograph that shows a bilateral sutural cataract

Sutural cataract is uncommon crystalline opacities that affect Y-sutures of the fetal lens nucleus, which is usually congenital and hereditary with X-linked transmission. We report the clinical observation of a 26-year-old patient with no significant pathological history, who consults for a decrease in bilateral progressive visual acuity. The ophthalmologic examination of both eyes found visual acuity at 8/10, the examen of the anterior segment found a clear cornea with Y-shaped crystalline opacities, the examen of the post segment is without abnormality (Figure 1). Therapeutically, the sutural cataract is not very progressive, and rarely require intervention because the effects on the vision are minimal. An optical correction by telescope was prescribed to this patient, with an improvement of the visual acuity at the level of both eyes at 10/10.

